# Supplementary figures and images for: Unveiling a novel memory center in human brain: neurochemical identification of the nucleus incertus, a key pontine locus implicated in stress and neuropathology
Source: Biol Res. 2024 Jul 16;57:46. doi: 10.1186/s40659-024-00523-z (PMC11253401; doi:10.1186/s40659-024-00523-z)

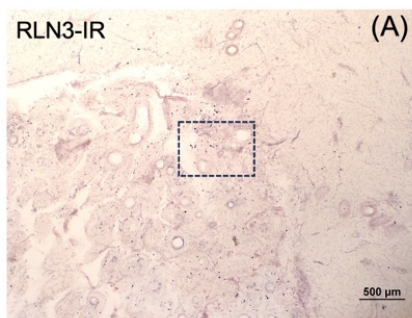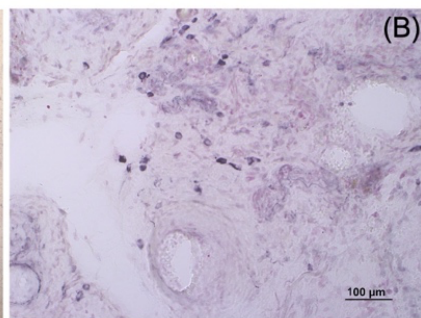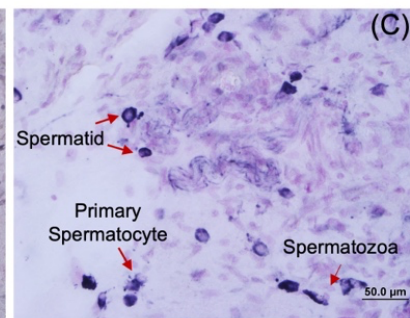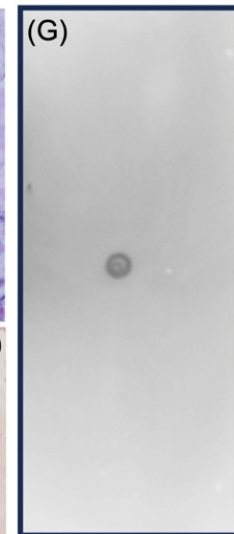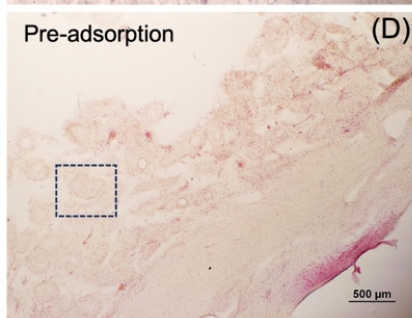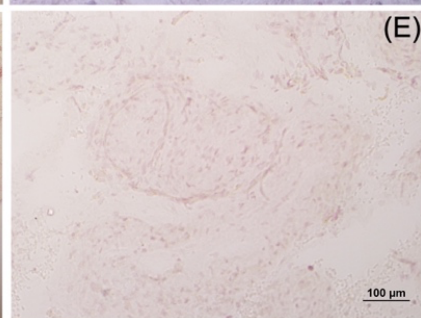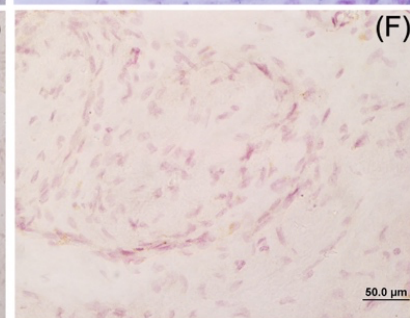

Supplement: Supplementary file 1 — Additional file 1. Fig. 1. Specificity of RLN3 Antiserum Assessed in Human Testis. (A-C) RLN3-immunoreactivity (IR) detected in spermatozoa cells in the human testis; (B,C) Higher magnification images of RLN3-IR in boxed area in A. (D-F) Pre-adsorption of RLN3 antibody pre-incubated overnight with RLN3 recombinant peptide. (E,F) Higher magnification images of boxed area in D. Tissue counterstained with neutral red. (G) Positive dot-blot for RLN3 antiserum against native peptide; circular immunoreactivity in the center of the cellulose membrane reflects the binding of the peptide (RLN3) to the antibody (anti-RLN3). [file 40659_2024_523_MOESM1_ESM.pdf]

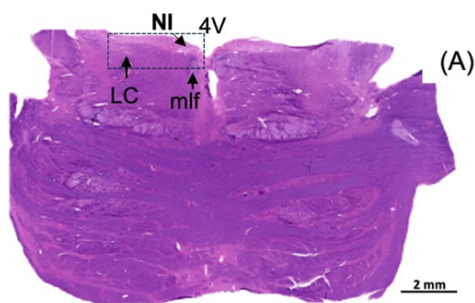

(A)

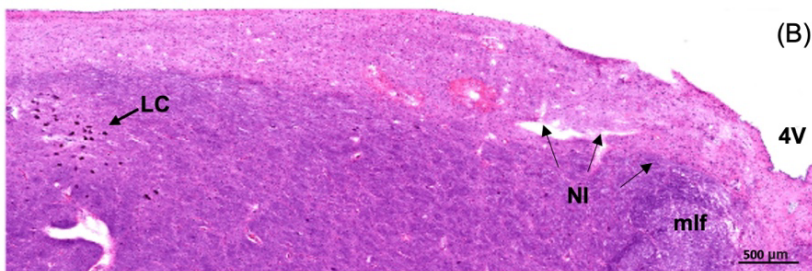

(B)

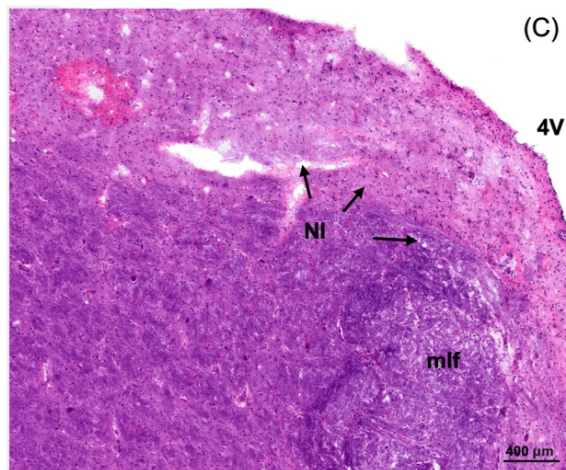

(C)

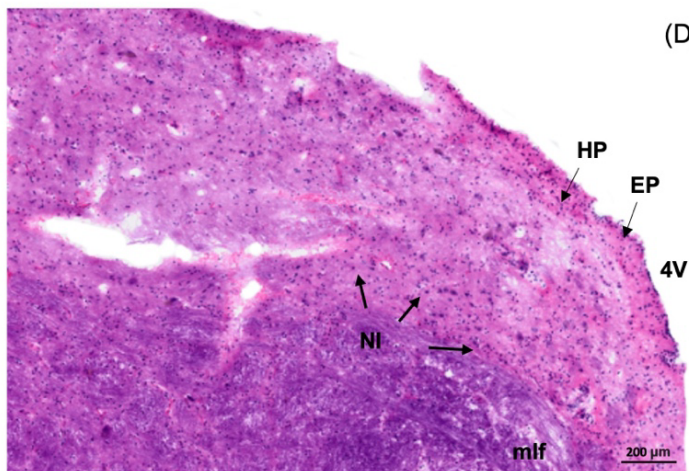

(D)

Supplement: Supplementary file 2 — Additional file 2. Fig. 2. Hematoxylin and Eosin Staining of Pons of Human Brain. (A) Low-power image of the neuroanatomy of the coronal pons containing the NI. (B) Higher magnification of the boxed area in A. (C) Higher magnification of the boxed area in B. (D) Higher magnification of the boxed area in C. Abbreviations: EP, ependyma, 4V, fourth ventricle, HG, Hypocellular gap, LC, locus coeruleus, mlf, medial longitudinal fasciculus, NI, nucleus incertus. [file 40659_2024_523_MOESM2_ESM.pdf]
